# Nano-Hydroxyapatite/Poly(methyl methacrylate) Composite Bone Scaffold: Surfactant Surface Effects

**DOI:** 10.3390/polym17091148

**Published:** 2025-04-23

**Authors:** Muhammed Enes Oruc, Nilüfer Evcimen Duygulu, Betul Onder, Aslihan Yelkenci, Cem Bülent Ustündag, Fatih Ciftci

**Affiliations:** 1 Department of Chemical Engineering, University of Doha for Science and Technology, Doha 24449, Qatar; muhammed.oruc@udst.edu.qa; 2 Department of Metallurgical and Material Engineering, Faculty of Chemical and Metallurgical Engineering, Yildiz Technical University, Istanbul 34210, Turkey; nevci@yildiz.edu.tr (N.E.D.); betul.onder@std.yildiz.edu.tr (B.O.); 3 Department of Pediatric Dentistry, Faculty of Dentistry, University of Health Sciences, Istanbul 34668, Turkey; aslihanzihni@gmail.com; 4 Department of Bioengineering, Faculty of Chemical and Metallurgical Engineering, Yıldız Technical University, Istanbul 34210, Turkey; cbustun@yildiz.edu.tr; 5 Health Biotechnology Joint Research and Application Center of Excellence, Istanbul 34210, Turkey; 6 Department of Biomedical Engineering, Fatih Sultan Mehmet Vakif University, Istanbul 34015, Turkey; 7 BioriginAI Research Group, Department of Biomedical Engineering, Fatih Sultan Mehmet Vakif University, Istanbul 34015, Turkey

**Keywords:** bone scaffold, hydroxyapatite nanomaterial, poly(methyl methacrylate), surfactant

## Abstract

In this study, poly(methyl methacrylate) (PMMA) nanofiber scaffolds reinforced with synthesized nano-hydroxyapatite (n-HA) were fabricated through electrospinning to enhance their potential for applications in bone tissue engineering. Sodium tripolyphosphate (STTP) was utilized as a surfactant to achieve a uniform distribution of particles and improve the structural integrity of the scaffolds. PMMA solutions were prepared at concentrations of the addition of STTP effectively stabilized n-HA dispersion, leading to enhanced fiber morphology, as confirmed by scanning electron microscopy (SEM), energy dispersive spectroscopy (EDS), and transmission electron microscopy (TEM). The PMMA_10_HA_S nanofibers demonstrated a homogeneous fiber distribution with an average diameter of 345.40 ± 53.55 nm and a calcium content of 7.1%. Mechanical testing revealed that adding STTP enhanced the mechanical properties, with the n-HA-reinforced 10 wt.% PMMA nanofibers achieving a maximum tensile stress of 4.16 ± 2.13 MPa and an elongation of 7.1 ± 1.95%. Furthermore, cell cytotoxicity assays of different concentrations (25, 50, 75, and 100 mg/mL) using L929 fibroblast cells demonstrated no cytotoxic effect of PMMA_10_HA_S nanofibers. These findings, reinforced by STTP and n-HA, highlight the potential of PMMA_10_HA_S nanofiber scaffolds as promising candidates for bone tissue applications.

## 1. Introduction

Scaffold architecture for bone regeneration has been the most popular approach in tissue engineering in the last decades [1,2,3]. The primary objective of scaffold-based bone tissue engineering is to create biomimetic structures that mimic the extracellular matrix (ECM) while supporting osteoblast adhesion, proliferation, and differentiation [4,5,6]. The interaction of osteoblasts with scaffolds has been extensively investigated, demonstrating that thinner nanofibers enhance cellular adhesion, proliferation, alkaline phosphatase activity, and ECM deposition [7,8]. The electrospinning technique is known for its simplicity and effectiveness in producing continuous fibers, enabling the fabrication of fibrous scaffolds with diameters ranging from nanometers to micrometers [9,10,11]. It has emerged as a promising technique for enhancing the properties of polymer-based scaffolds.

Among these polymers, PMMA is widely used in orthopedic applications and bone tissue engineering [12,13,14,15]. In orthopedic surgery, PMMA facilitates load transfer between prosthetic implants and bone structures [16,17,18]. Despite their widespread use, PMMA-based bone tissue materials have several limitations, including poor bioactivity and a lack of chemical bonding with bone tissue, which can lead to implant loosening and secondary fractures [19,20]. To overcome these challenges, it is essential to develop PMMA-based scaffolds that offer improved bioactivity and mechanical stability [21,22]. Many researchers have explored adding various filler materials or blending components into the solution to enhance the morphological, mechanical, and biological properties of PMMA-based scaffolds [21,23,24,25].

Hydroxyapatite (HA), the main mineral component of bone, has emerged as a promising bioactive agent because of its outstanding osteoconductive properties [26] and biocompatibility [27,28]. In the literature, MTT studies were performed on n-HA-reinforced PMMA nanocomposites on fibroblast cells, and no cytotoxic effects on cell viability were observed [29,30,31,32,33]. Nano-sized hydroxyapatite (n-HA) offers advantages such as a high surface area and suitability for integration into nanofibrous scaffolds via electrospinning techniques [34,35,36]. PMMA/n-HA nanocomposite fibers produced through electrospinning exhibit high surface area and nanoscale porosity, making them not only promising candidates for bone tissue engineering but also applicable in air filtration, volatile organic compound removal, and nanoparticle capture in industrial settings [15,36]. Incorporating n-HA into PMMA matrices has been shown to improve interfacial shear strength at the bone–implant interface and enhance osteoblast activity compared to PMMA [15,37,38,39]. However, achieving a uniform dispersion of n-HA particles within the PMMA matrix remains a significant challenge. The high surface energy of n-HA leads to agglomeration, which significantly diminishes its reinforcing efficiency and negatively affects the mechanical and biological properties of the resulting scaffold [37,38,39,40,41]. Additionally, the inherently porous and brittle structure of n-HA further limits its standalone clinical applicability.

Several advanced mixing and surface modification techniques have been explored to overcome these challenges of dispersion, such as ultrasonic dispersion, high-power sonication, dual asymmetric centrifugal (DAC) [41] mixing, and the use of dispersing agents such as poly(acrylic acid) (PAA) [37]. Other practical approaches include polymer grafting via surface-initiated atom transfer radical polymerization (ATRP) [42], hydrothermal synthesis, poly(ethylene oxide) (PEO) [43] coating, surface modification with heparin, and refinement through centrifugation. While these methods effectively improve dispersion quality, they often require complex procedures, specialized equipment, and increased processing time, limiting their scalability and practicality for widespread use.

Surfactants have gained attention as accessible and efficient alternatives due to their ability to reduce interfacial tension and stabilize nanoparticles through steric or electrostatic repulsion mechanisms. Among the various surfactants [44,45,46,47], sodium tripolyphosphate (STTP) is a promising candidate for dispersing n-HA. STTP is cost-effective, easy to implement, and has excellent binding affinity for calcium-rich surfaces such as hydroxyapatite. Numerous studies have explored the use of STTP in nanoparticle production. For instance, Zhang et al. [48] examined the impact of STTP addition on HA nanocrystals. However, there is a lack of research regarding incorporating STTP into polymer matrices and the effects on their properties.

Therefore, this study aims to develop electrospun PMMA/n-HA nanocomposite scaffolds incorporating STTP as a surfactant. The goal is to achieve a uniform distribution of particles and enhance the structural integrity with the inclusion of STTP. Structural characterization was conducted using X-ray diffraction (XRD), while morphological analyses were performed using scanning electron microscopy (SEM, TEM) and energy dispersive spectroscopy (EDS) mapping. The mechanical properties were evaluated through tensile testing, and biocompatibility was assessed via the XTT cell viability assay.

## 2. Experimental

### 2.1. Materials and Methods

For electrospinning of nanofibers, PMMA (Mw = 350 K Sigma-Aldrich, Darmstadt, Germany), dimethyl-formamide (DMF, Merck, Darmstadt, Germany), and tetrahydrofuran (THF, Merck) were used. For hydroxyapatite synthesis, calcium nitrate tetrahydrate (Ca (NO_3_)_2_·4H_2_O, Merck KGaA, Darmstadt, Germany), ammonium dihydrogen phosphate ((NH_4_) H_2_PO_4_, Merck, KGaA, Darmstadt, Germany) and ammonia solution (NH_4_OH, 28%, Merck Co. Darmstadt, Germany) were used. As the surfactant, sodium tripolyphosphate (STTP, Sigma-Aldrich, Darmstadt, Germany) was selected.

### 2.2. Preparation of n-HA

PMMA (Mw = 350 K) (Sigma-Aldrich), tetrahydrofuran (THF, Merck), and dimethyl-formamide (DMF, Merck) were employed. To synthesize hydroxyapatite, ammonium dihydrogen phosphate ((NH_4_) H_2_PO_4_, Merck, KGaA, Darmstadt, Germany), calcium nitrate tetrahydrate (Ca (NO_3_)_2_·4H_2_O, Merck Co. Darmstadt, Germany), and ammonia solution (NH_4_OH, 28%, Merck Co. Darmstadt, Germany) were utilized. The amount of starting materials for n-HA synthesis was determined as a Ca/P ratio of 1.67; 23.66 g of Ca(NO_3_)_2_·4H_2_O were dissolved in 100 mL of water. The prepared Ca(NO_3_)_2_·4H_2_O and (NH_4_)_2_HPO_4_ were mixed in an ultrasonic bath for 15 min. A stock solution was prepared by dissolving 7.93 g of (NH_4_)_2_HPO_4_ in 100 mL of water. The pH of the prepared solutions was adjusted to pH ≥ 10 with a drop of NH_4_OH to bring the pH ≥ 10. Prepared Ca(NO_3_)_2_·4H_2_O and (NH_4_)_2_ HPO_4_ stock solutions were used at a volume of 20 mL each and mixed dropwise. To precipitate, the resulting nano-sized hydroxyapatite crystals were kept at room temperature for 24 h. The crystals were washed 6 times at 4100 rpm for 5 min to remove the residues formed due to the reaction [49,50].

### 2.3. Electrospinning of PMMA Nanofibers

The electrospinning solution was prepared by dissolving PMMA in a binary solvent of DMF and THF at a 1:1 ratio under constant stirring for 24 h. Then, 0.5 wt.% n-HA particles (designated as PMMA_HA) and STTP (0.3 wt.% of n-HA) (designated as PMMA_HA_S) were added to the solution and mixed for 2 h. The prepared solutions were subsequently transferred into plastic syringes fitted with 15-gauge needles. A distance of 15 cm was maintained between the spinneret and the collector plate, which was covered with aluminum foil. A DC voltage of 10 kV was applied, and the feed rate was set to 0.5 mL/h.

### 2.4. Morphological Characterization

The morphological structures of n-HA and PMMA_HA fibers were examined using scanning electron microscopy (SEM; JEOL, Tokyo, Japan). Energy dispersive spectrometry (EDS) was performed using the Oxford Instruments, Abingdon, UK system and INCA Suite Version 4.09, Oxford Instruments, Abingdon, UK. A transmission electron microscope (JEOL JEM 2100, Tokyo, Japan) was utilized to analyze the particle size and distribution. The surfaces of the samples were coated with platinum using a sputter coater (Polaron SC7640, Quorum Technologies, Newhaven, UK). The investigations were conducted at a 10 kV accelerating voltage. JEOL and ImageJ 13.0.6. software (U.S. National Institutes of Health, Bethesda, MD, USA) software were used to measure the fiber diameters and their size distributions. For phase characterization of the synthesized n-HA particles, an X-ray diffractometer (XRD, Rigaku, RINT 2200 VL, Tokyo, Japan) was utilized with CuKα radiation.

### 2.5. Mechanical Analysis

The mechanical properties of the fibers were assessed through a tensile mechanical test, following the standard procedure outlined in ASTM D882 [51,52]. The samples were processed into rectangular strips with 100 mm × 20 mm × 0.1 mm dimensions. Tensile strength and strain tests were conducted using a tensile tester (Shimadzu—EZ-LX, Tokyo, Japan) and specialized TRAPEZIUM LITE X software 1.0. All samples were tested at a 5 mm/min speed until they reached their breaking point. The measurements were taken at room temperature (23 °C).

### 2.6. Cell Viability XTT Test

Bone scaffold samples were separated from the greaseproof paper and placed on 2 × 2 microplates. L929 cells were then seeded into each well of 48-well flat-bottom microplates at a concentration of 3 × 10^4^ in 500 µL of medium. The cells were incubated at 37 °C for 24 h to allow for cell attachment. After the 24-h incubation period, the entire volume of the culture medium was aspirated. Then, 500 µL of fresh medium containing 5-carboxanilide (XTT) at a concentration of 7.5 mg/mL, along with 0.5 mg/mL of phenazine methosulfate and 2,3-bis-(2-methoxy-4-nitro)-5-sulfophenyl-2H-tetrazolium, was added to each well. The cells were incubated for an additional 4 h at 37 °C. Cell culture medium was used as a negative control. Percent cell viability was calculated by measuring the optical density at 450 nm.

The effect of different medium volumes (25 mg/mL, 50 mg/mL, 75 mg/mL, 100 mg/mL) on cell viability was tested on L929 cells seeded into 96-well microplates. Bone scaffold samples were placed in a cell culture medium for 24 h. After a 24-h incubation period, a standard TXT test was performed, and cell viability was calculated and visualized as a percentage.

### 2.7. Statistical Analysis

All statistical data analyses were conducted using ANOVA with GraphPad Prism version 8 software (GraphPad Software Inc., San Diego, CA, USA). The values are presented as means ± standard deviation (SD), and statistical differences were analyzed by one-way ANOVA and Tukey and Dunnett multiple comparison tests. In all instances, *p* < 0.05 was deemed statistically significant.

## 3. Results and Discussion

### 3.1. Characterization of HA Nanoparticles

**Figure 1:**
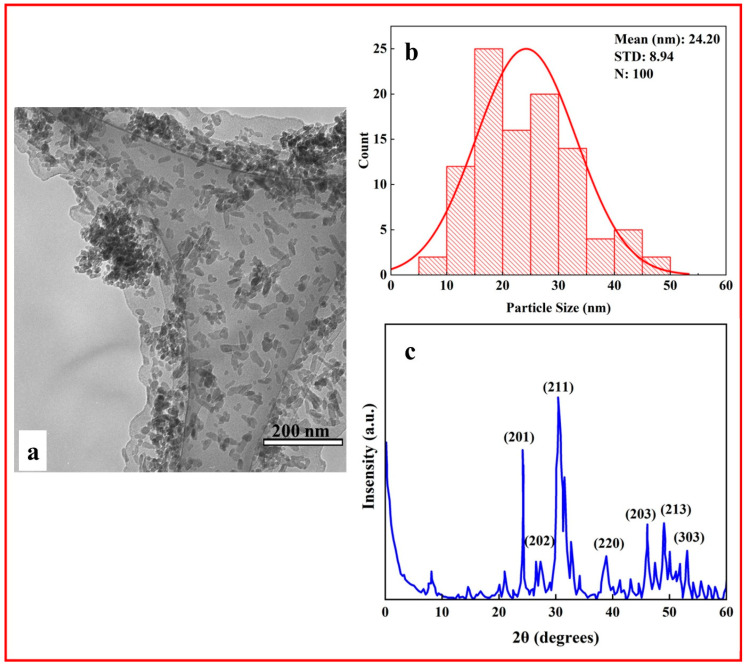
(**a**) TEM, (**b**) particle size distribution histogram, (**c**) XRD pattern of n-HA.

The synthesis of HA nanoparticles was based on our previous study as a reference. [53]. Figure 1 shows the TEM analysis (a) and particle size distribution of n-HA (b). The average n-Ha is measured as 24 ± 8 nm. The X-ray diffraction patterns of n-HA depict that all the peaks correspond to stoichiometric HA (JSPDS Card no. 09-432) (Figure 1c).

### 3.2. Morphological Analysis of PMMA Nanofibers

**Figure 2:**
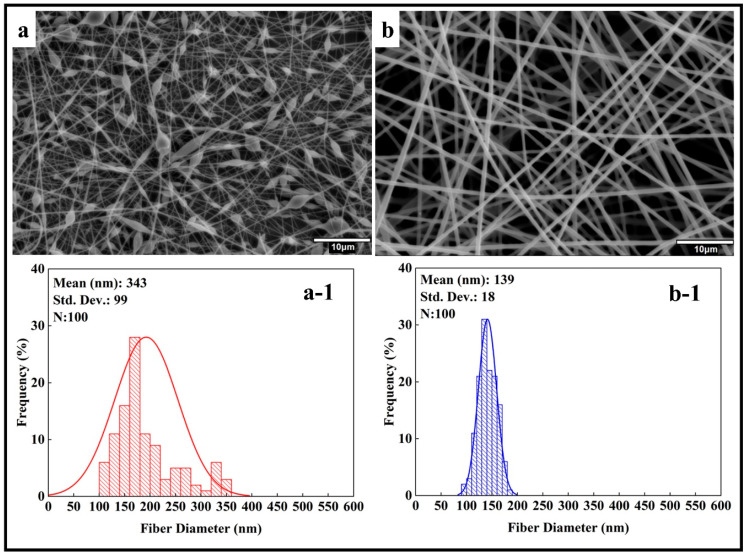
SEM images and histogram diagrams of PMMA nanofibers at different concentrations (wt.%): (**a**,**a-1**) 5, (**b**,**b-1**) 10.

In the current study, the concentration of PMMA varied from 5 wt.% to 10 wt.% while maintaining a constant solvent ratio of DMF/THF (1:1). Figure 2 shows the SEM images and histograms of the PMMA nanofibers. Bead formation was observed at a 5 wt.% PMMA concentration (PMMA_5). In contrast, at a 10 wt.% PMMA concentration (PMMA_10), bead-free fibers were produced. The average fiber diameter for the PMMA_10 sample was measured at 139 ± 18 nm. When excluding beads from the calculation for the PMMA_5 sample, the average fiber diameter was 343 ± 99 nm. The presence of beads at lower concentrations can be attributed to insufficient polymer concentration to form uniform and smooth fibers at a specific molecular weight. The concentration of polymers greatly affects the fiber diameter and quality during the electrospinning process [54]. As the polymer concentration increases, the fiber diameter tends to increase while the formation of beads decreases [55,56]. DMF is commonly used as a solvent for PMMA electrospinning, and assisting THF enhances the conductivity of the solution [57]. DMF has a boiling point of 153 °C, whereas THF has a boiling point of 66 °C [58,59]. During the electrospinning process, THF evaporates quickly due to its relatively low boiling point, which can promote the formation of droplets and the development of porous fiber structures. In contrast, DMF has a higher boiling point, so it was added to the solution to improve stability. Additionally, the presence of DMF in the PMMA_10 solution may result in slower evaporation, potentially causing stretching during jet formation and contributing to a reduction in fiber diameter [7,60].

When considering the diameter of nanofibers, lower concentrations are advantageous due to the larger surface area they provide for applications in tissue engineering and filtration, as surface area plays a crucial role in cell attachment and the adsorption of contaminant particles. [61,62,63]. Also, the bead-on-a-string structure (PMMA_5) can be avoided by using other low-surface tension solvents, increasing the viscosity of the polymer solution by assisting high viscous solvents or reducing the feed rate of the polymer [64].

**Figure 3:**
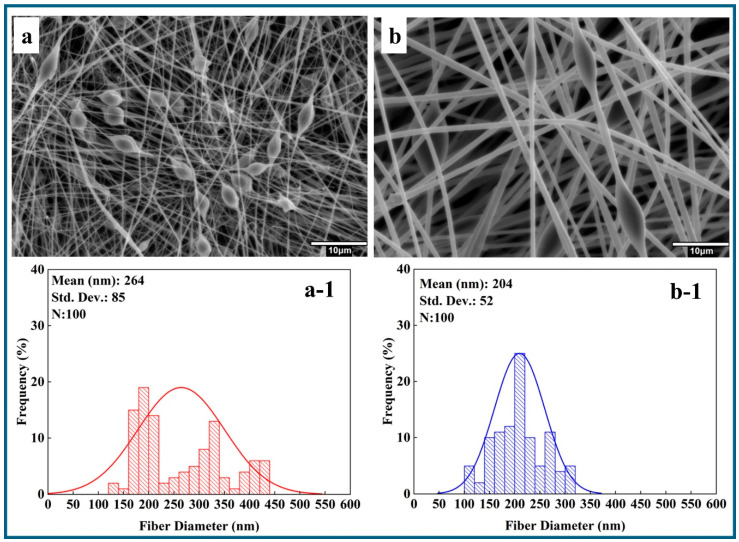
SEM images and histogram diagrams of n-HA blended PMMA nanofibers at different PMMA concentrations (wt.%): (**a**,**a-1**) 5, (**b**,**b-1**) 10.

Hydroxyapatite, known for its porous structure, plays a vital role in bone tissue. However, it has certain limitations in biomedical applications. To overcome these challenges, HA was blended with PMMA. Research has been conducted to examine how HA nanoparticles disperse within electrospun PMMA nanofibers. The concentration of 0.5 wt.% of n-HA was blended into the PMMA solutions. Figure 3a,b display SEM images and corresponding histograms (Figure 3(a-1,b-1)) of the electrospun PMMA_HA nanofibers. According to the histogram data, the fiber diameters measured were 264 ± 85 nm for PMMA_5_HA (excluding beads from the measurement) and 204 ± 52 nm for PMMA_10_HA.

However, SEM observations indicated that the blending of n-HA resulted in precipitation issues in the PMMA_5 and PMMA_10 nanofibers. This precipitation may diminish the effectiveness of n-HA, particularly in biomedical applications.

**Figure 4:**
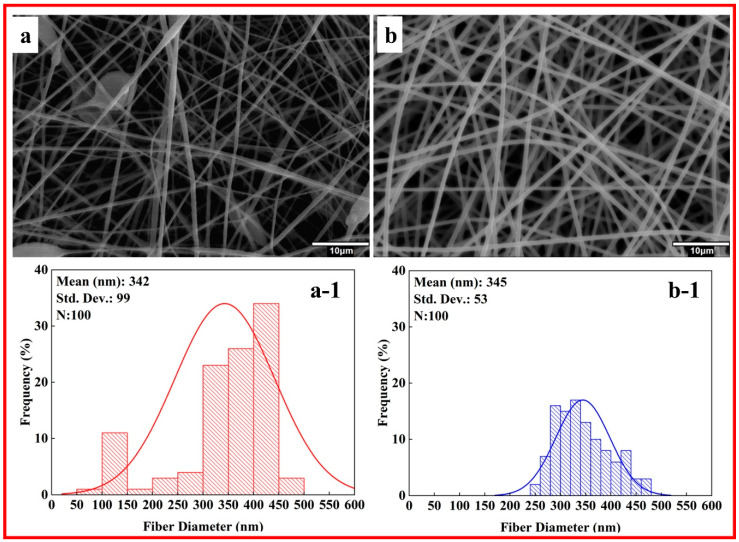
SEM images and histogram diagrams of n-HA blended PMMA nanofibers with surfactant addition at different PMMA concentrations (wt.%): (**a**,**a-1**) 5, (**b**,**b-1**) 10.

Surfactants are molecules with hydrophilic and hydrophobic domains, allowing them to interact with various materials. The current study added STTP to the PMMA_HA solutions to address this issue. In Figure 4, SEM and the histograms of PMMA_HA nanofibers are given after surfactant additions. According to SEM images after surfactant addition, the stability of the HA dispersion in the solvent was achieved, and the average fiber diameters were measured as 342 ± 99 and 345 ± 53 nm for PMMA_5_HA_S and PMMA_10_HA_S, respectively. In the PMMA_HA system, the surfactant positions itself at the boundary between the hydrophilic n-HA particles and the hydrophobic PMMA matrix. This action decreases the interfacial tension and establishes a protective steric or electrostatic layer around the n-HA particles. As a result, the surfactant enhances the stability of the n-HA particles in suspension, effectively minimizing aggregation and preventing sedimentation.

**Figure 5:**
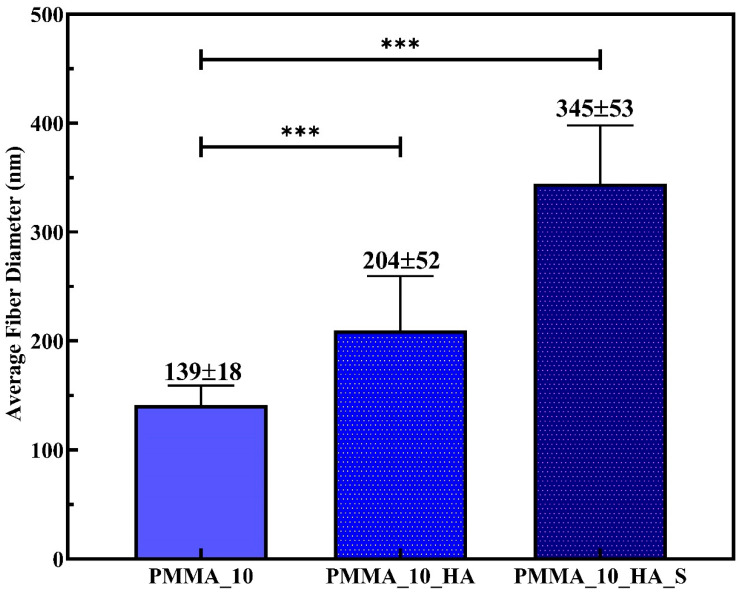
Comparison of fibers diameters. Relationship between average fiber diameters of PMMA, PMMA_HA, and PMMA_HA_S nanofibers. (Relative mean expression indicates relative expression *** *p* < 0.001).

Bead formation issues were observed in all PMMA_5, PMMA_5_HA, and PMMA_5_HA_S nanofibers. In contrast, no bead formation problems were detected in the PMMA_10 samples. Moreover, the addition of a surfactant improved the dispersion of n-HA. In Figure 5, the bar graph displays the average fiber diameters of three electrospun nanofibers: PMMA_10, PMMA_10_HA, and PMMA_10_HA_S. Statistically significant differences between the groups are indicated by asterisks (***), signifying a high level of significance (*p* < 0.001). The average diameters measured for the PMMA_10, PMMA_10_HA, and PMMA_10_HA_S composite nanofibers were 139 ± 18 nm, 204 ± 52 nm, and 345 ± 53 nm, respectively.

The results indicate that the average fiber diameter of PMMA_10 increased with the addition of n-HA reinforcement. Similar findings were reported by Sheikh et al. [65], who noted that the increased viscosity due to the presence of HAp nanoparticles led to the formation of larger droplets. This caused significant bending instability during fiber formation, increasing nanofiber diameters. STPP improves the dispersion of PMMA/HA nanofibers through a combination of surface modification, electrostatic stabilization, and morphology control. It adsorbs onto the surface of HA particles, particularly at active crystal growth sites, altering their growth behavior and reducing agglomeration [48,66]. Zhang et al. [48] observed that STPP affects particle size and shape, increasing the diameter of particles. Furthermore, STPP introduces negative surface charges, promoting electrostatic repulsion between particles and preventing clustering [67].

In this study, a low concentration of STPP was utilized, making the adsorption effect likely more significant than any changes in ionic strength or solubility. Our observations revealed that adding surfactants increased the average fiber diameter. Surfactants can modify the surface tension and viscosity of the solution, potentially leading to larger fiber diameters. They can also improve the dispersion of n-HA particles, which might initially seem to reduce fiber diameter [68,69]. However, the overall effect on viscosity and surface tension can still result in thicker fibers.

**Figure 6:**
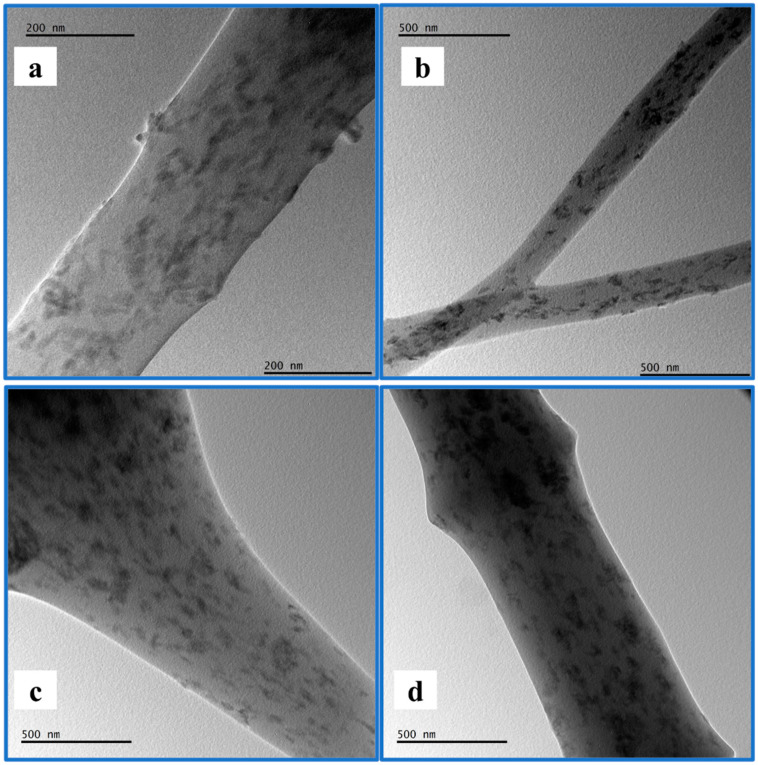
TEM image of (**a**) PMMA_5_HA, (**b**) PMMA_5_HA_S, (**c**) PMMA_10_HA, (**d**) PMMA_10_HA_S.

Xing et al.’s [70] research indicated that higher HA content enhances the adhesion of osteoblasts on electrospun fiber mats. Furthermore, HA is an excellent adsorbent material for decontaminating various pollutants from water due to its high sorption capacity for metal ions [71]. This capacity arises from the presence of P-OH groups on the surface of HA, which act as adsorption sites for metallic ions [72]. The electrospun fiber mat was modified with n-HA, enhancing its suitability for environmental applications. Additionally, TEM characterization of the PMMA fibers containing n-HA was performed, as illustrated in Figure 6. The TEM images show that adding a surfactant significantly enhances the dispersion of n-HA particles within the PMMA nanofiber. This enhancement reduces particle aggregation and encourages uniformity, especially at higher PMMA concentrations (10 wt.%). Without a surfactant, n-HA particles often cluster, particularly in denser matrices. Therefore, incorporating a surfactant is vital for achieving well-integrated and homogeneous nanofibers, ultimately enhancing the mechanical and bioactive properties of the composite for biomedical applications.

**Figure 7:**
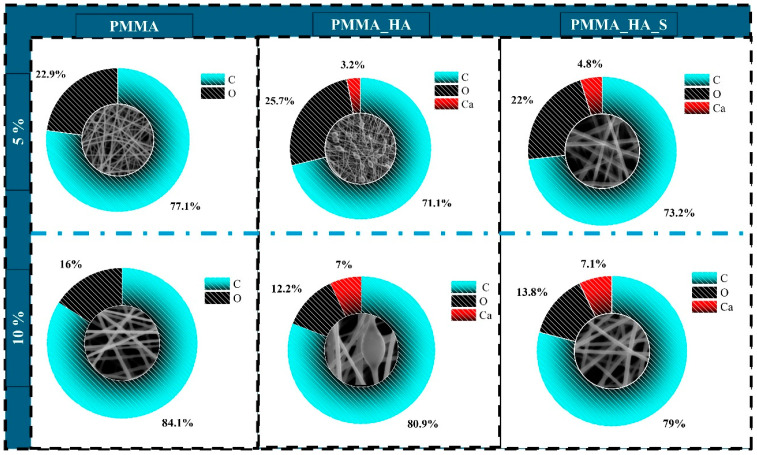
EDS analysis of PMMA nanofibers.

Figure 7 presents the SEM-EDS analysis of PMMA nanofibers, confirming the presence of HA nanoparticles through the detection of calcium (Ca) along with the primary elements of PMMA: carbon (C) and oxygen (O). Also, the surfactant addition improved the dispersion of the n-HA particles, resulting in a higher percentage of calcium. The highest concentration of Ca, 7.1%, was observed in the sample labelled PMMA_10_HA_S.

### 3.3. Mechanical Analysis of PMMA Nanofibers

**Figure 8:**
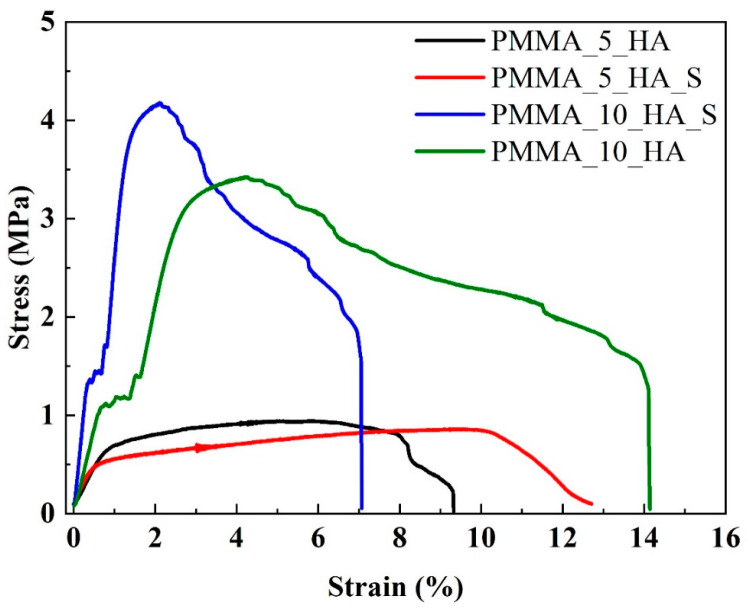
Tensile stress–strain curves of PMMA_5_HA, PMMA_5_HA_S, PMMA_10_HA, and PMMA_10_HA_S nanofibers.

Figure 8 presents the results of the tensile tests as a stress–strain curve. The strength value was determined by identifying the maximum stress, while the strain at maximum stress was calculated based on the elongation. Additionally, the slope of the elastic region was assessed, as shown in Table 1. The stress–strain curves indicate that adding a surfactant significantly enhances the dispersion of HA by reducing nanoparticle agglomeration and promoting better interfacial adhesion between HA and the PMMA matrix. This effect is particularly noticeable in the PMMA_10_HA_S sample, demonstrating the best balance between mechanical strength and ductility. These results underscore the synergistic influence of HA content and surfactant addition in improving the mechanical behavior of the composite.

The tensile test results indicate that the PMMA_10_HA sample exhibits a tensile strength of 3.42 MPa and a maximum elongation value of 14.2%. In contrast, the PMMA_5_HA_S sample exhibits the lowest strength, at 0.85 MPa, and a moderate elongation of 12.7%. As the surfactant is added to HA, strength and elongation increase in the PMMA_10 samples.

### 3.4. Cell Viability XTT Analysis of PMMA Nanofibers

**Figure 9:**
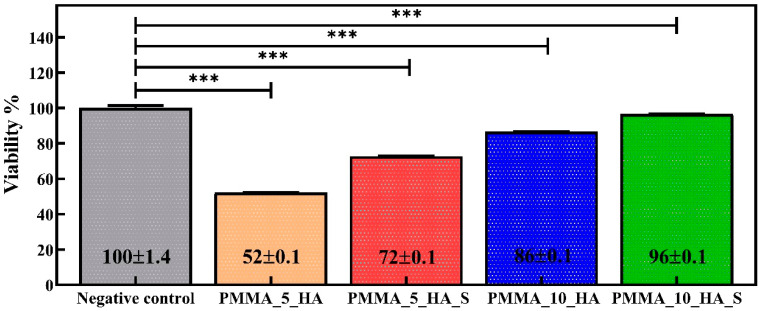
Cell viability results are given as % of the negative control, mean ± SD values of *n* = 3 independent experiments. *** *p* < 0.001 vs. negative control group. Data were analyzed using one-way analysis of variance (ANOVA) and the Tukey test.

The cell viability data in Figure 9 demonstrates the cytocompatibility of PMMA_HA composite nanofibers containing surfactant and without STPP. The viability percentages are expressed relative to the negative control (100%). Statistically significant differences are indicated, with *** *p* < 0.001 based on one-way ANOVA and Tukey’s post hoc analysis. The PMMA_5_HA composite nanofibers markedly reduced cell viability by 52%, while the addition of STPP nanofibers significantly improved cell viability by 72%.

The PMMA_10_HA composite fibers without the surfactant achieved 86% cell viability. Notably, the PMMA_10_HA_S composite nanofiber attained the highest viability, reaching 96%. This increase can be attributed to the effect of the surfactant, which enhances the biocompatibility of materials. The presence of the surfactant allows cells to interact more effectively with the material’s surface by eliminating hydrophobic regions and increasing surface energy. Additionally, the addition of STTP demonstrated enhanced biocompatibility in the composite materials, improving cell viability and promoting cell adhesion to the material’s surface. The cell viability performance of PMMA fibers is consistent with their applications in dentistry and orthopedics [73,74,75].

**Figure 10:**
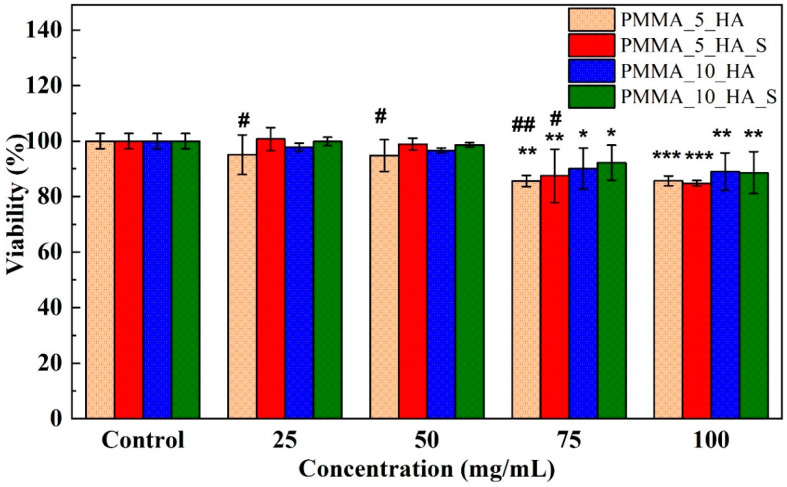
Cell viability of L929 cells exposed to different concentrations (25, 50, 75, and 100 mg/mL) of PMMA nanofibers. Data represents standard deviations (*n* = 3), * *p* < 0 05, ** *p* < 0.01, and *** *p* < 0.001 vs. control group; ^#^ *p* < 0.05, and ^##^ *p* < 0.01 vs. PMMA nanofibers. Data were analyzed using one-way analysis of variance (ANOVA) and the Dunnett test.

The cytotoxicity study indicates the effects of various concentrations of materials on cell viability (Figure 10). It shows that all materials tested exhibit low toxicity and are compatible with biological systems. The cell viability values for all materials generally ranged from 80% to 100% across the concentration range of 25 mg/mL to 100 mg/mL. This suggests that the materials do not display a significant concentration-dependent cytotoxic effect. PMMA fibers demonstrated stable performance at all concentrations and exhibited no toxic effects. This finding further supports the bioinert properties of PMMA. In HA-doped fibers, it is observed that cell viability slightly increases as the doping ratio rises. PMMA_10_HA composite fibers exhibited a high viability rate at all concentrations, emphasizing the beneficial effects of HA on bone regeneration and cell proliferation. Surfactant-added fibers tend to enhance cell viability at both additive ratios. The PMMA_10_HA_S composite fibers exhibit the highest viability percentage across all concentration ranges. This implies that surfactants improve cell adhesion and metabolism on the material surface. Surfactants may facilitate cellular proliferation by promoting direct contact between the material and the cells.

In related studies, an MTT cell viability test was conducted on PMMA_HA and two-dimensional magnesium phosphate (MgP) nanofibers for orthopedic applications. It was noted that HA-doped PMMA/MgP nanofibers, exhibiting high bioactivity, were the most suitable for promoting cell viability in the nanocomposite [76]. Gonçalves et al. [77] compared fibroblasts from L929 with Saos-2 osteoblasts on PMMA_HA and graphene oxide scaffolds. The fibroblasts demonstrated 91% cell viability and a 0.5% apoptosis rate, while the Saos-2 osteoblasts showed 100% viability and a 1.9% apoptosis rate.

Several studies have examined the biocompatibility of HA/PMMA nanocomposites for temporary dental restorations. For instance, Zhang et al. [32] found that composites with 40% HA promoted cell proliferation in human gingival fibroblasts comparable to titanium. MTT assays showed that when fibroblasts were cultured on PMMA discs with 20–50% HA, the 40% HA composites achieved similar proliferation rates to pure titanium. At the same time, lower HA concentrations reduced cell growth [32]. Shirdar et al. [78] carried out a study to optimize the mechanical properties and cytocompatibility of PMMA nanocomposites reinforced with HA nanofibers and magnesium phosphate nanosheets. The MTT assay was performed using 3T3-J2 fibroblast cells to evaluate cell viability. The results showed that the 7.5% HA group exhibited the highest cell viability and mechanical strength, with no cytotoxic effects observed [78].

Additionally, S. Kalidas et al. [29] performed an MTT assay on L929 mouse fibroblast cells using nanocomposites from PMMA doped with n-HA. The results showed that scaffolds containing 60% HAp exhibited high cell proliferation, and no cytotoxic effects were detected. The incorporation of HAp significantly enhanced biocompatibility [29].

## 4. Conclusions

The electrospinning technique was employed to fabricate PMMA- and n-HA-reinforced nanofibers. PMMA solutions were prepared using THF and DMF as solvents, while n-HA was synthesized to form a composite structure with PMMA. A surfactant was added to the PMMA_n-HA to achieve a uniform nanofiber morphology. The surfactant effectively reduced the interfacial tension between the hydrophilic n-HA particles and the hydrophobic PMMA matrix, thereby influencing the diameters of the fibers. Morphological analyses revealed that the average fiber diameter for PMMA_5_HA_S was 342.80 ± 99.01 nm, whereas for PMMA_10_HA_S, the average diameter was 345.40 ± 53.55 nm. The addition of STTP enhanced the stability of n-HA particles in the solvent, reducing aggregation and preventing precipitation. The mechanical tests indicated that the tensile strength of PMMA_5_HA_S nanofiber decreased to 0.85 ± 1.20 MPa, while the elongation ratio increased to 12.7 ± 2.73%. In the case of PMMA_10_HA_S fibers, the tensile strength rose to 4.16 ± 2.13 MPa with the addition of surfactant, though the elongation ratio fell to 7.1 ± 1.95%.

Regarding biocompatibility, the PMMA_10_HA_S composite nanofibers displayed the % cell viability, reaching 96%. This increase is attributed to the surfactant increasing the biocompatibility of the material. Additionally, the PMMA_5_HA_S nanofibers increased cell viability to 72% upon the addition of STTP. In contrast, the PMMA_10_HA composite nanofibers without surfactants demonstrated a % cell viability of 82%, suggesting that adding surfactants positively influences cell adhesion and proliferation. Cytotoxicity assessments confirmed that all tested fibers maintained cell viability between 80% and 100% across the 25 mg/mL to 100 mg/mL concentration range, with no significant concentration-dependent toxic effects observed.

Furthermore, a slight increase in cell viability was observed in HA-doped nanofiber groups as the HA content rose. Notably, PMMA_10_HA composite nanofibers consistently showed high cell viability across all tested concentrations, underscoring the beneficial role of HA in promoting bone regeneration and cellular activity proliferation. These findings confirm the cellular compatibility and potential biomedical applicability of PMMA/n-HA composite nanofibers, particularly with surfactant incorporation, making them suitable for tissue engineering and bone regeneration applications.

## Figures and Tables

**Table 1:** Tensile test results (* Statistical significance level was determined as *p* < 0.05, and the parenthesis indicates standard deviation).

Sample Code	Stress (MPa)	Elongation (%)
PMMA_5_HA	0.95 ± 1.45 *	9.3 ± 2.24 *
PMMA_5_HA_S	0.85 ± 1.20 *	12.7 ± 2.73 *
PMMA_10_HA	3.42 ± 1.56 *	14.2 ± 2.87 *
PMMA_10_HA_S	4.16 ± 2.13 *	7.1 ± 1.95 *

## Data Availability

The authors confirm that the data supporting the findings of this study are available within this article. Raw data that support the findings of this study are available from the corresponding author, upon reasonable request.
